# dRYBP Counteracts Chromatin-Dependent Activation and Repression of Transcription

**DOI:** 10.1371/journal.pone.0113255

**Published:** 2014-11-21

**Authors:** Sol Fereres, Rocío Simón, Adone Mohd-Sarip, C. Peter Verrijzer, Ana Busturia

**Affiliations:** 1 Centro de Biología Molecular “Severo Ochoa” CSIC-UAM, c) Nicolás Cabrera 1, 28049 Madrid, Spain; 2 Department of Biochemistry and Center for Biomedical Genetics, Erasmus University Medical Center, Wytemaweg 80, 3015 CN, Rotterdam, The Netherlands; Università degli Studi di Milano, Italy

## Abstract

Chromatin dependent activation and repression of transcription is regulated by the histone modifying enzymatic activities of the trithorax (trxG) and Polycomb (PcG) proteins. To investigate the mechanisms underlying their mutual antagonistic activities we analyzed the function of *Drosophila* dRYBP, a conserved PcG- and trxG-associated protein. We show that dRYBP is itself ubiquitylated and binds ubiquitylated proteins. Additionally we show that dRYBP maintains H2A monoubiquitylation, H3K4 monomethylation and H3K36 dimethylation levels and does not affect H3K27 trimethylation levels. Further we show that dRYBP interacts with the repressive SCE and dKDM2 proteins as well as the activating dBRE1 protein. Analysis of homeotic phenotypes and post-translationally modified histones levels show that dRYBP antagonizes dKDM2 and dBRE1 functions by respectively preventing H3K36me2 demethylation and H2B monoubiquitylation. Interestingly, our results show that inactivation of dBRE1 produces trithorax-like related homeotic transformations, suggesting that dBRE1 functions in the regulation of homeotic genes expression. Our findings indicate that dRYBP regulates morphogenesis by counteracting transcriptional repression and activation. Thus, they suggest that dRYBP may participate in the epigenetic plasticity important during normal and pathological development.

## Introduction

The dynamic and reversible chromatin modifications mediated by the evolutionary conserved Polycomb (PcG) and trithorax (trxG) proteins contribute to the maintenance of repressed and active transcriptional states [1]. Although great progress has been made in deciphering the biochemical activities of the PcG/trxG, little is known about the interplay between PcG-mediated repression and trxG-mediated activation. PcG/trxG regulate the expression of numerous genes, the best known being the Hox genes. *Drosophila* Hox genes contain PREs/TREs (Polycomb and Trithorax Response elements) that serve as platforms for the recruitment of the PcG/trxG protein complexes [1]. Binding of PcG/trxG proteins at these sites leads to transcriptional regulation via post-translational modification of histones [2],[3]. These modifications can have antagonistic effects on transcription. For example, the monoubiquitylation of histone H2A (H2Aub) mediated by the E3-ubiquitin ligase SCE/dRING promotes transcriptional repression [4],[5] while the monoubiquitylation of histone H2B (H2Bub) mediated by the E3-ubiquitin ligase dBRE1 promotes transcriptional activation [6]. The dBRE1 protein forms a complex with RAD6 [7] which is required to promote H2B ubiquitylation, a prerequisite for the H3K4 methylation that promotes transcriptional activation. SCE/dRING is a core subunit of the repressor PRC1 complex that also contains Polycomb (PC), Posterior Sex Comb (PSC) and Polyhomeotic (PH). Knowledge of the compositional diversity of PRC1 is expanding [1] and variants of PRC1 and non-canonical PRC1 complexes with distinct transcriptional outcomes have been isolated [8]–[10]. For example, the dRAF complex (dRing Associated Factors) composed of dRING, PSC (both members of canonical PRC1) and dKDM2 promotes stronger repression than PRC1 as it stimulates monoubiquitylation of H2A more efficiently and also demethylates H3K36me2, a modification established by trxG [11]. Moreover, recent results in vertebrates indicate the existence of non-canonical PRC1 complexes that, instead of containing the core subunit PC (CBX in vertebrates) they contain the RYBP subunit forming the PRC1-RYBP complexes found to locate at target genes with intermediate levels of expression [8],[9]. The finding of non-canonical PRC1 complexes has led to recent discoveries that are challenging the classical hierarchical recruitment complex model whereby PRC1 complex is recruited by PRC2-mediated H3K27 trimethylation [12]. It has now been shown in vertebrates that non-canonical PRC1-mediated H2A monoubiquitylation can recruit PRC2 [13]–[16].

The conserved dRYBP/YAF2/RYBP protein contains in its N-terminal a Ubiquitin Binding Domain (UBD) of the type Nucleoporin Zinc Finger (NZF) and the murine RYBP has been shown to interact with ubiquitin [17]–[19]. Loss of dRYBP function in *Drosophila* produces a range of phenotypes that are highly variable in penetrance [19],[20] suggesting that dRYBP functions in a range of biological processes. Moreover, although dRYBP inactivation does not produce homeotic phenotypes, dRYBP has been shown to interact with PcG/trxG proteins and to function as a PcG-dependent transcriptional repressor [19],[20]. However, the mechanisms underlying dRYBP function in epigenetic regulation of gene expression mediated by PcG/trxG proteins remain poorly understood.

Here we show that dRYBP interacts genetically and biochemically with dRING, dKDM2 and dBRE1 to modulate H2Aub, H3K36me2 and H2Bub levels and thereby regulate gene repression and activation.

## Results and Discussion

### dRYBP interacts with ubiquitin and with ubiquitylated proteins

**Figure 1:**
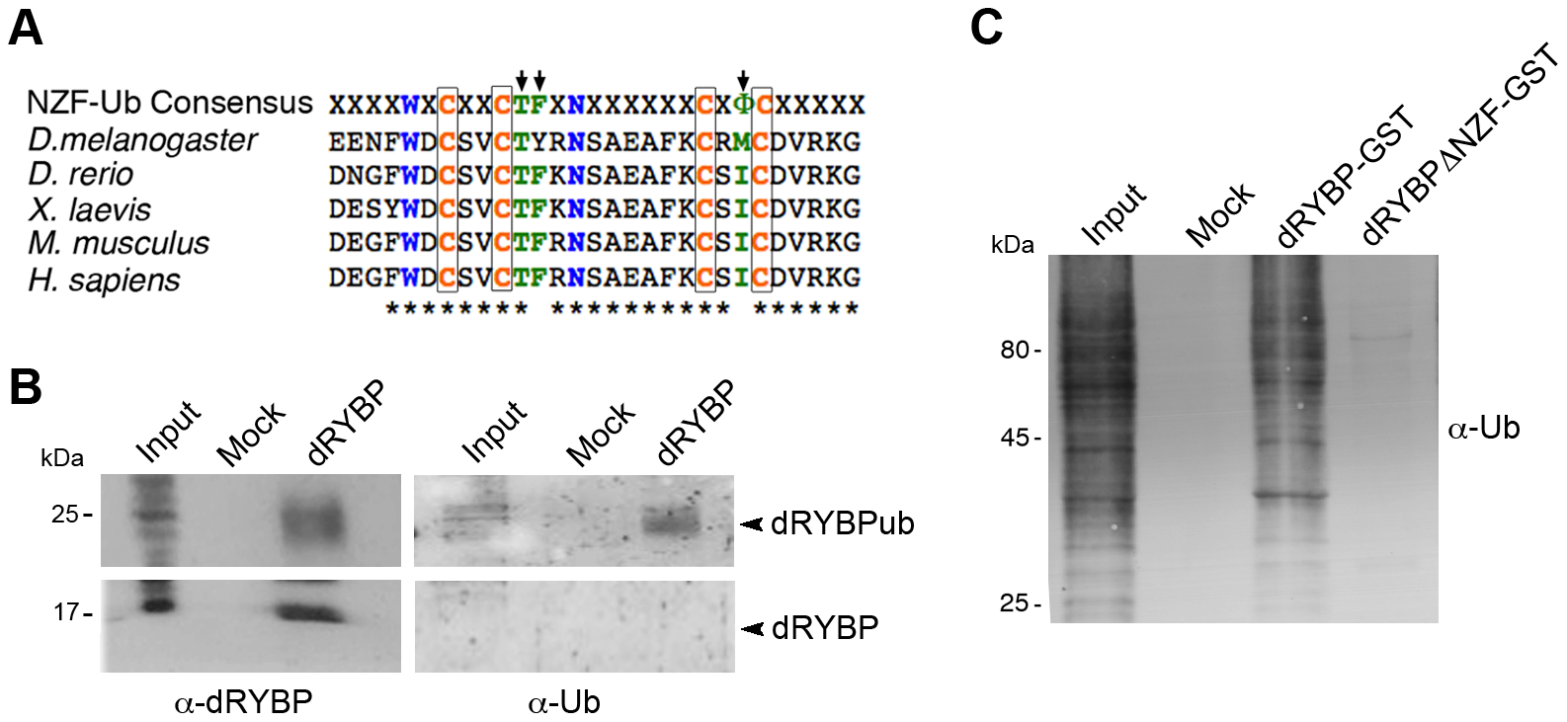
dRYBP binds to ubiquitin and ubiquitylated proteins. (**A**) Phylogenetic conservation of the dRYBP protein consensus NZF domain. Indicated are the aminoacids required for Zn binding (orange), for protein folding (blue) and for Ub binding (arrows, green). (**B**) Western Blot of immunoprecipitation using α-dRYBP antibody of *Drosophila* embryonic nuclear protein extracts for dRYBP and Ub detection. Input: *Drosophila* embryonic nuclear protein extracts. Mock: pre-immune serum. Indicated are the 17 kDa and 25 kDa bands corresponding to dRYBP and dRYBPub. (**C**) Western Blot detection with α-Ub antibody of dRYBP-GST or dRYBPΔNZF-GST-pulldowns from S2 cell extracts treated with proteasome inhibitors (+MG-132+Lact.). Input: S2 cell extracts. Mock: protein-GST.

The dRYBP protein sequence (Figure 1A) suggests its function in the process of ubiquitylation [17],[18], a crucial step in the epigenetic regulation of transcription [2],[21]. We first analyzed whether dRYBP binds ubiquitin. We performed immunoprecipitation of *Drosophila* wild type nuclear protein extracts using anti-dRYBP antibody and the samples were analyzed by immunoblotting with anti-ubiquitin and anti-dRYBP antibodies. Figure 1B shows that the anti-dRYBP antibody detects a protein band of 17 kDa (corresponding to dRYBP) and another of 25 kDa while the anti-ubiquitin antibody only detects the 25 kDa band. Thus, dRYBP coexists in *Drosophila* in two different forms: dRYBP and dRYBPub.

Next, we analyzed whether dRYBP interacts with ubiquitylated proteins. We generated fusion proteins with full length dRYBP (dRYBP-GST) and a truncated form of the dRYBP protein lacking the UBD domain (dRYBPΔNZF-GST). These fusion proteins were used to perform GST pulldowns with S2 cells protein extracts treated with proteasome inhibitors (Figure 1C) to increase the abundance of ubiquitylated proteins in the extracts (Figure S1). The pulldown products were analyzed by immunoblotting using the anti-ubiquitin antibody. Results show that the bulk of the ubiquitylated proteins were found to bind to dRYBP-GST (Figure 1C). Thus, the UBD domain of the dRYBP protein seems to be required for the majority of the interactions, in S2 cells, between dRYBP and ubiquitylated proteins.

This behavior of *Drosophila* dRYBP protein is similar to the murine RYBP [18]. dRYBP coexists as two different forms, dRYBP and dRYBPub proteins and interacts with ubiquitylated proteins (Figure 1). Functions of the two protein forms are yet to be determined. Our results show that dRYBP monoubiquitylation does not induce its proteasomal degradation (Figure S1). We speculate that dRYBP and, in particular dRYBPub form could serve as adaptor proteins to facilitate the assembly of ubiquitylation complexes and thus, promote protein ubiquitylation. Additionally, the interaction of dRYBP, through its NZF domain, with many of the ubiquitylated proteins present in S2 cells (Figure 1C) could explain the diverse biological processes in which dRYBP is involved such as apoptosis, immune response and morphogenesis [19],[22]–[24] as well as the variety of dRYBP/RYBP interactor proteins so far described including, the E2F transcription factor, Apoptin and Hungtintin [25],[26].

### The dRYBP protein associates with SCE, dKDM2 and dBRE1

**Figure 2:**
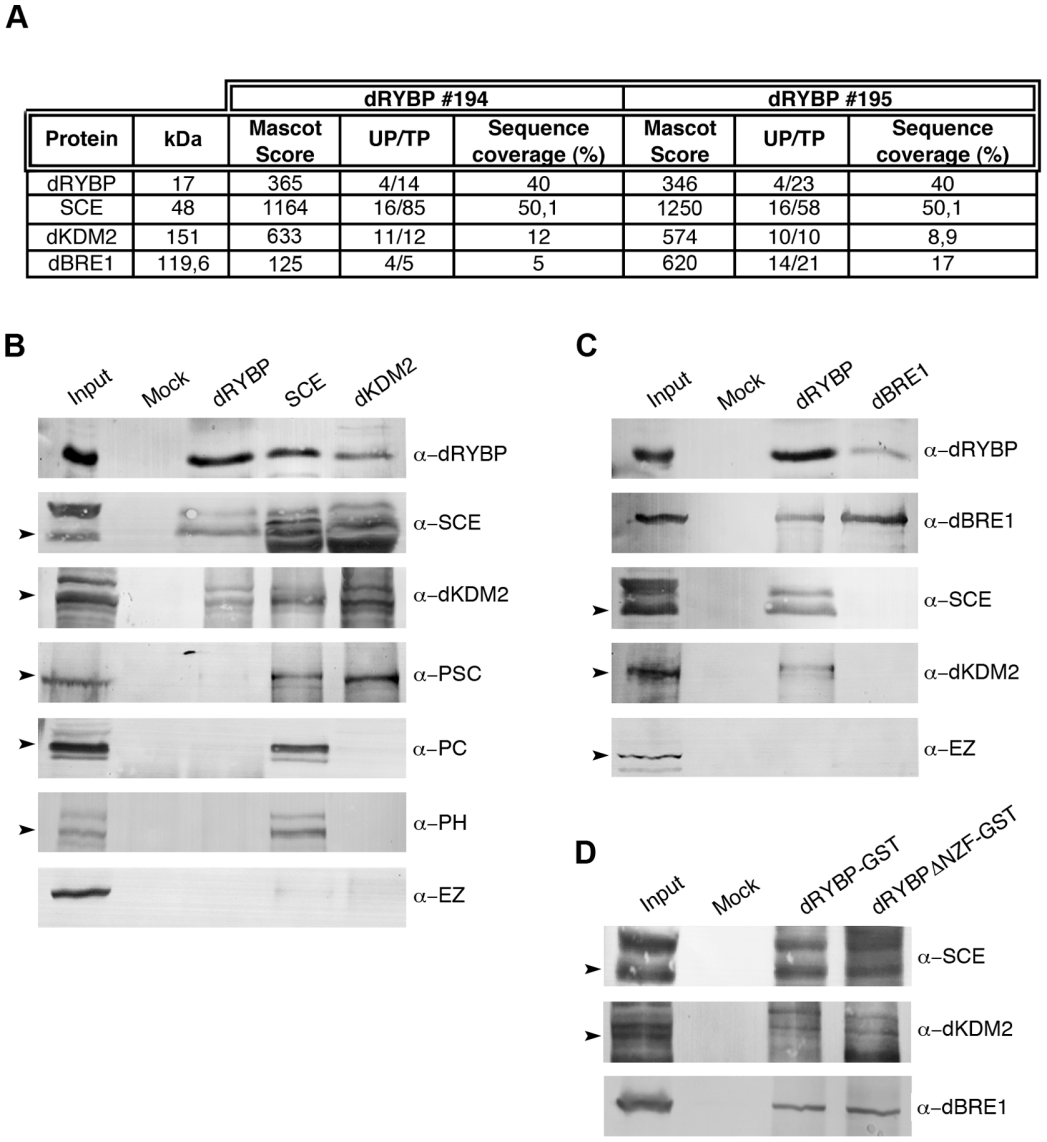
dRYBP interacts biochemically with SCE, dKDM2 and dBRE1. (**A**) Mass spectrometric parameters of the dRYBP, SCE, dKDM2 and dBRE1 proteins using *Drosophila* embryonic nuclear protein extracts and dRYBP #194 and dRYBP #195 serum antibodies. Molecular weight (kDa), mascot score (MS), number of unique peptides (UP), total number of peptides (TP) identified and sequence coverage (%). (**B**) Western Blot of co-immunoprecipitations using α-dRYBP, α-SCE or α-dKDM2 antibodies for dRYBP, SCE, dKDM2, PSC, PC, PH and E(Z) detection. (**C**) Western Blot of co-immunoprecipitations using α-dRYBP or α-dBRE1 antibodies for dRYBP, dBRE1, SCE, dKDM2 and E(Z) detection. In (B) and (C) *Drosophila* embryonic nuclear protein extracts (Input), pre-immune serum (Mock). (**D**) Western Blot detection with the indicated antibodies of dRYBP-GST or dRYBPΔNZF-GST-pulldowns using *Drosophila* embryonic nuclear protein extracts (Input) or protein-GST (Mock). Arrowheads point to the corresponding protein bands detected with the indicated antibodies (other bands may be non-specific or correspond to modified proteins).

We used two different dRYBP affinity purified serum antibodies to isolate dRYBP and its interacting proteins from nuclear protein extracts of 0 to 12 hour-old *Drosophila* embryos. Immunoprecipitation was followed by extensive washes with a buffer containing either 400 mM or 800 mM of KCl and 0,1% of NP-40 (see Materials and Methods). Mass spectrometric analysis revealed a large number of dRYBP-associated proteins, among these SCE, dKDM2 and dBRE1 (Figure 2A). Curiously any other PcG proteins such as PSC, SU(Z)2, PH, PC or PHO were detected at either 400 mM or 800 mM of KCl.

We confirmed the association of dRYBP with SCE, dKDM2 and dBRE1 by co-immunoprecipitation (co-IPs) experiments of *Drosophila* nuclear protein extracts combined with immunoblotting using anti-dRYBP, anti-SCE, anti-dKDM2 and anti-dBRE1 antibodies (Figure 2). Additionally, we analyzed the interaction of dRYBP with other PcG proteins such as PSC, PC and PH that did not appear as dRYBP interacting proteins in our analysis but have been shown to form complexes with dRING/SCE and/or dKDM2 (Figure 2B). As a negative control for all proteins we checked for association with E(Z) protein [27] (Figure 2B). Co-IPs of dRYBP, SCE, dKDM2, dBRE1 showed that dRYBP is associated with SCE, dKDM2 and dBRE1 (Figure 2B and Figure 2C) and that dRYBP does not associate with PSC, PC, PH and E(Z) (Figure 2B). Additionally, co-IPs also show that dBRE1 does not interact with SCE or dKDM2 (Figure 2C). Reverse co-IPs confirmed these interactions (Figure S2). Of note, the co-IP experiments only detected the interaction between PcG/trxG proteins with the unmodified dRYBP protein of 17 kDa. Future experiments will investigate whether the dRYBPub protein (25 kDa) can also interact with SCE, dKDM2 and dBRE1 proteins as a way to understand the functions of both protein forms.

To analyze whether the UBD domain of dRYBP is required for its association with SCE, dKDM2 and dBRE1 we performed pulldown assays using the dRYBP-GST and dRYBPΔNZF-GST proteins with *Drosophila* nuclear protein extracts. Results show that both dRYBP-GST and dRYBPΔNZF-GST associate with SCE, dKDM2 and dBRE1 proteins (Figure 2D) indicating that the UBD domain of dRYBP is not required for these interactions. Therefore, the NZF domain of the dRYBP protein is perhaps essential for dRYBP functions independent of its interaction with PcG/trxG proteins and therefore independent of its function as chromatin-dependent transcriptional regulator.

Thus, the analysis of the dRYBP interactions with the subunits of the vertebrate non-canonical RYBP-PRC1 (RYBP, PSC, dRING/SCE) and canonical PRC1 complexes (PC, PSC, dRING/SCE and PH) indicates that dRYBP only interacts with dRING/SCE (Figure 2B). Therefore, it seems that the subunit composition of the non-canonical RYBP-PRC1 complexes may not be conserved between flies and mammals and perhaps, in *Drosophila*, the dRYBP protein may impede the direct interaction between dRING/SCE and PSC [28],[29]. Also, the vertebrate BCOR complex containing RYBP [30],[31] is considered the equivalent of the *Drosophila* dRAF complex. Therefore, it has been generally assumed that dRAF also contains dRYBP [1]. However, this is not the case as our results show that dRYBP only interacts with the SCE/dRING and dKDM2 subunits of the dRAF complex (Figure 2). These results lead us to propose the existence of a dRRK (dRING, dRYBP, dKDM2) complex, a variant of the dRAF complex that excludes PSC. Finally, little is known about the dBRE1 interactor proteins. So far, the only protein reported to interact with BRE1 is RAD6 [7]. However, RAD6 was not detected as a dRYBP interacting protein in the mass spectrometric analysis. Our results lead us to propose the existence of a dRB complex consisting of dBRE1 and dRYBP. The dRRK and dRB complexes may have distinct effects on gene expression.

### *dRYBP* genetically interacts with *Sce*, *dkdm2* and *dBre1*

**Figure 3:**
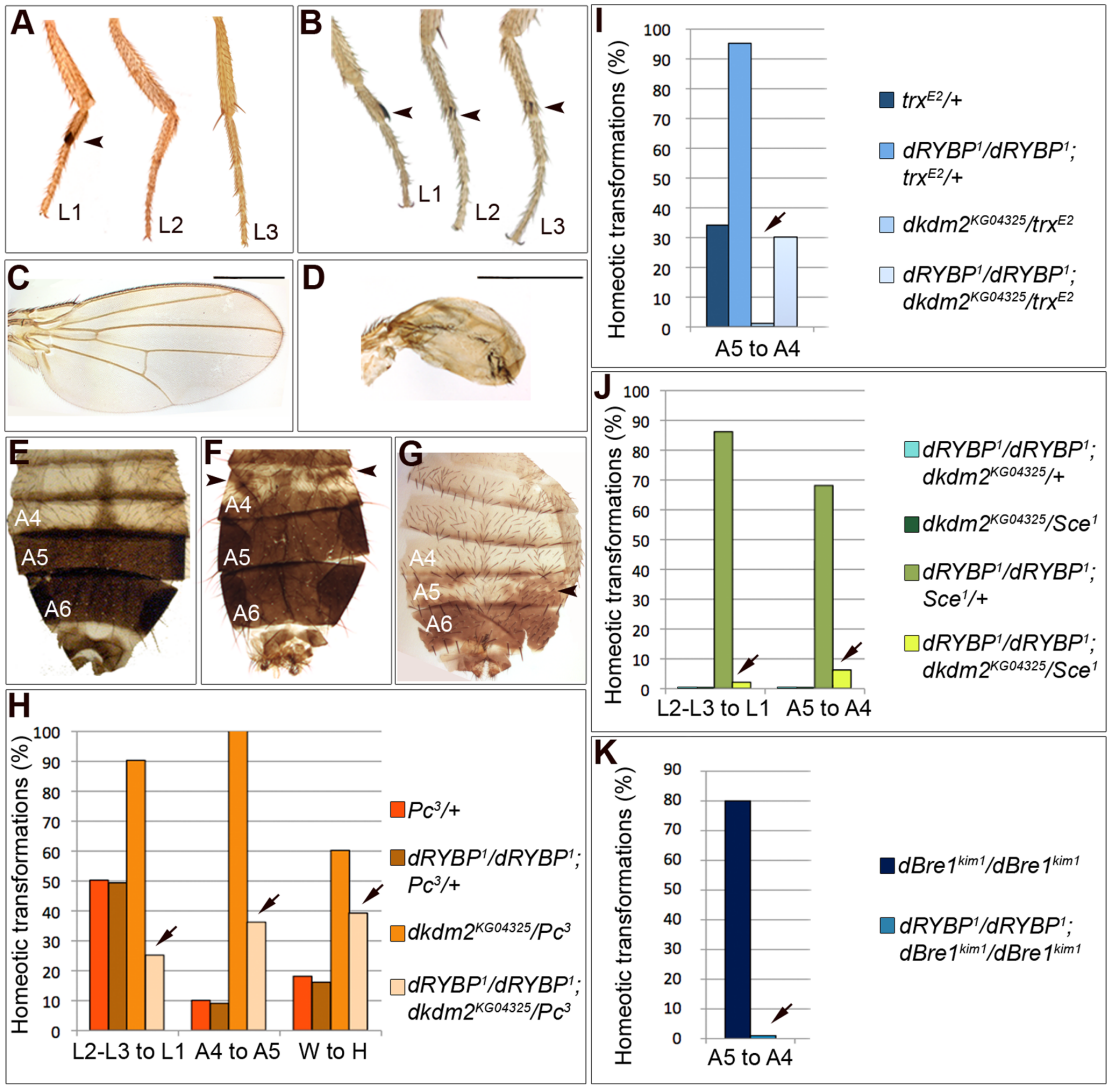
*dRYBP* interacts genetically with *Sce*, *dkdm2* and *dBre1*. (**A**) Wild type male legs (L1, L2 and L3). L1 presents the sex comb (arrowhead) not present in L2 or L3. (**B**) *dRYBP^1^/dRYBP^1^; Pc^3^/dkdm2^KG04325^* legs showing ectopic sex combs on L2 and L3 (arrowheads). (**C**) Wild type wing. (**D**) *dRYBP^1^/dRYBP^1^; Pc^3^/dkdm2^KG04325^* wing partially transformed to haltere. Scale bars represent 200 µm. (**E**) Wild type male abdomen. Indicated are the A4, A5 and A6 segments. Note the pigmentation of A5 and A6. (**F**) *dRYBP^1^/dRYBP^1^; Pc^3^/dkdm2^KG04325^* male abdomen. Note the patches of pigmentation (arrowheads) in A4. (**G**) *dRYBP^1^/dRYBP^1^; trx^E2^*/+ male abdomen showing patches of de-pigmentation (arrowhead) in the A5. (**H-K**) Graphs representing the frequency of the indicated phenotypes in flies of the indicated genotypes. (**H**) Genetic interaction between *dRYBP* and *dkdm2* in a *Pc* mutant background. Arrows mark the frequency of the indicated phenotypes in *dRYBP^1^/dRYBP^1^*; *dkdm2^KG04325^/Pc^3^* flies (n = 100 in all cases). (**I**) Genetic interaction between *dRYBP* and *dkdm2* in a *trx* mutant background. Arrows mark the frequency of the indicated phenotypes in *dRYBP^1^/dRYBP^1^*; *dkdm2^KG04325^/trx^E2^* flies (n = 100 for all genotypes). **(J**) Genetic interaction between *dRYBP*, *dkdm2* and *Sce* (n = 100 for *dRYBP^1^/dRYBP^1^*; *dkdm2^KG04325/^*+ and *dRYBP^1^/dRYBP^1^*; *Sce^1^/+*; n = 45 for *Sce^1^/dkdm2^KG04325^*; n = 70 for *dRYBP^1^/dRYBP^1^*; *Sce^1^/dkdm2^KG04325^*). Arrows mark the frequency of the *dRYBP^1^/dRYBP^1^*; *Sce^1^/dkdm2^KG04325^*. (**K**) Genetic interaction between *dRYBP* and *dBre1*. Arrow marks the frequency of *dRYBP^1^/dRYBP^1^*; *dBre1^kim1^/dBre1^kim1^* (n = 100 in all cases).

We hypothesized that *dRYBP* genetically interacts with *Sce* and *dkdm2* to control gene silencing and with *dBre1* to control gene activation. To test this, we analyzed the *PcG* genes inactivation related homeotic phenotypes such as: the transformation of meta- (L3) and meso-thoracic (L2) legs into pro-thoracic (L1) legs (L2-L3 to L1, Figure 3A, B), the transformation of wings into halteres (W to H, Figure 3C, D) and the transformation of the forth (A4) abdominal segment towards the fifth (A5) one in males (A4 to A5, Figure 3 E, F). Furthermore, we analyzed the *trxG* genes inactivation related homeotic phenotype such as the transformation of segment A5 towards segment A4 (A5 to A4, Figure 3E, G).

We first determined the penetrance of these homeotic phenotypes in *dRYBP* and *dkdm2* double mutant flies. Neither loss of *dRYBP* function [20] nor loss of *dkdm2* function [11] or the double mutant combination (i.e., *dRYBP^-^/dRYBP^-^; dkdm2^-^/dkdm2^-^*, this study) show homeotic phenotypes. Therefore, we investigated the interaction phenotypes in flies with a sensitized mutant genetic background *i.e.* in *Polycomb* heterozygous mutant flies (*Pc^3^/+*) and in *trithorax* heterozygous mutant flies (*trx^E2^/+*) flies. The use of these sensitized mutant genetic backgrounds has been previously probed very useful to detect interactions between *PcG* and *trxG* genes [20],[32]–[34].

*Polycomb* heterozygous mutant flies (*Pc^3^/+*) present *PcG*-related mutant homeotic phenotypes (50% L3-L2 to L1; 18% W to H; 10% A4 to A5, Figure 3H). Flies *dkdm2^KG04325^/Pc^3^* [11] show an increase in the penetrance of these phenotypes (90% L3-L2 to L1; 60% W to H; 100% A4 to A5), suggesting that *dkdm2* is an enhancer of *Pc* [11]. When *dRYBP* and *dkdm2* are concomitantly inactivated (*dRYBP^1^/dRYBP^1^*; *dkdm2^KG04325^/Pc^3^*, Figure 3H) the penetrance of the phenotypes was significantly reduced (26% L3-L2 to L1; 38% W to H; 39% A4 to A5, Figure 3H) suggesting that *dRYBP* is a suppressor of *dkdm2* repressor effect.

Moreover, *trithorax* heterozygous mutant flies (*trx^E2^/+)* present the A5 to A4 transformation with 33% penetrance (Figure 3I) and this frequency is highly increased in the absence of *dRYBP* function (94% of the *dRYBP^1^/dRYBP^1^*; *trx^E2^/+*, Figure 3I) indicating that *dRYBP* is an enhancer of *trx* [20]. Conversely, the frequency of the A5 to A4 phenotype of *trx^E2^/+* is decreased in the absence of *dkdm2* function (2% of *dkdm2^KG04325^/trx^E2^*, Figure 3I) indicating that *dkdm2* is a suppressor of *trx* [11]. When, *dRYBP* and *dkdm2* are simultaneously inactivated (*dRYBP^1^/dRYBP^1^; dkdm2^KG04325^/trx^E2^*), the *trx* enhancer effect of *dRYBP* decreases (from 94% to 31%) and the *trx* suppressor effect of *dkdm2* decreases (from 2% to 31%). Thus, these results indicate that *dRYBP* and *dkdm2* antagonize each other activities.

Next, we analyzed the genetic interactions with *Sce*. The heterozygous *Sce^1^/+* mutant flies show neither *PcG*- nor *trxG*-related homeotic phenotypes ([35],[36] and Figure 3J) even when *dkdm2* expression is reduced (*Sce^1^/dkdm2^KG04325^*, Figure 3J). However, the simultaneous inactivation of *dRYBP* and *Sce* (*dRYBP^1^/dRYBP^1^*; *Sce^1^/+*) produces both *PcG*- and *trxG-*related homeotic phenotypes (L2–L3 to L1 with a 86% penetrance and A5 to A4 with a 68% penetrance, Figure 3J) suggesting that *dRYBP* and *Sce* are enhancers of *Pc* and, interestingly that *dRYBP* and *Sce* are enhancers of *trx* [20]. Notably, the frequency of both of these phenotypes is significantly decreased (2% L2-L3 to L1 and 6% A5 to A4, Figure 3J) when the levels of *dkdm2* expression are reduced (*dRYBP^1^/dRYBP^1^*; *Sce^1^/dkdm2^KG04325^*). Therefore, *dkdm2* is a suppressor of the *dRYBP* and *Sce* repressor effects (from 86% to 2%) and of the *dRYBP* and *Sce* enhancer effects (from 68% to 6%).

Finally, we studied the genetic interaction between *dRYBP* and *dBre1*. As indicated in Figure 3K, *dBre1^kim1^* homozygous mutant flies (*dBre1^kim1^*/*dBre1^kim1^*) show the A5 to A4 transformation with high frequency (80%, Figure 3K). However, in the absence of *dRYBP* (*dRYBP^1^/dRYBP^1^*; *dBre1^kim1^*/*dBre1^kim1^*) there is a significant decrease in the frequency (2%) of the A5 to A4 transformation, suggesting that *dRYBP* suppresses the activator function of *dBre1*.

The results from our study of the genetic interaction of *dRYBP*, *dkdm2* and *Sce*, indicate that dRYBP associates with these proteins to counteract and alleviate the dKDM2-mediated transcriptional repression. Additionally, the study of the the genetic interaction between *dBre1* and *dRYBP* show, for the first time, the function of dBRE1 as a trxG protein in the regulation of homeotic gene expression and indicate that dRYBP associates with dBRE1 protein to counteract and alleviate the dBRE1-mediated activation.

### dRYBP modulates levels of H2Aub, H2Bub and H3K36me2

Our biochemical and functional results showing interactions of *dRYBP* with *dkdm2*, *Sce* and *dBre1* raised the possibility that the ability of *dRYBP* to counteract both transcriptional repression and activation is a result of the modulation of post-translational histones modifications. To investigate this possibility, we focused the analysis on the levels of H2Aub, H3K36me2 and H2Bub as these are the main targets of dKDM2, SCE and dBRE1 [4],[11],[37]. We also studied H3K27me3 levels to investigate the dRYBP role on PRC2-mediated recruitment in *Drosophila* [12],[14].

**Figure 4:**
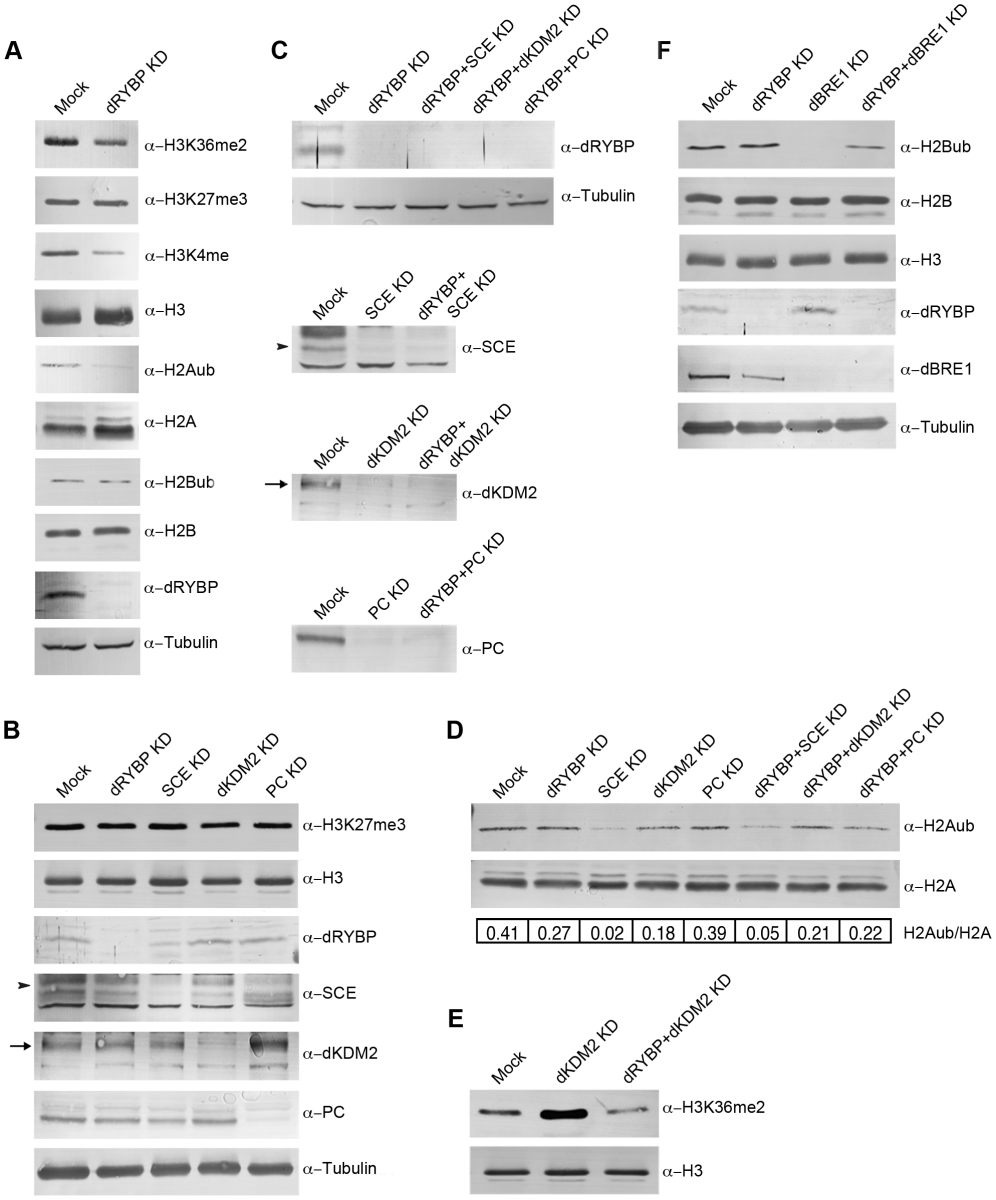
dRYBP inactivation modulates levels of histone modifications. Western Blot analysis of whole S2 cells histone extracts or protein extracts to analyze either levels of histone modifications or protein levels to control dsRNA-inactivation efficiency with the indicated antibodies. The reduction (%) of expression was calculated measuring and quantifying the intensity of the bands using Fiji imaging software and Tubulin expression as a reference. (**A**) Inactivation of dRYBP. Note the slight decrease of H3K36me2 and strong decrease of both H3K4me and H2Aub and the efficiency of the dRYBP inactivation (98%). (**B**) Levels of H3K27me3 in the indicated KDs. Note that H3K27me3 levels do not change and the efficiency of the inactivation (dRYBP 78%, SCE: 66%, dKDM2: 90%, PC: 56%). (**C**) Levels of the indicated proteins in the corresponding KDs. Efficiency of dRYBP reduction in dRYBP KD: 97%; in dRYBP + SCE KD: 98%; in dRYBP + dKDM2 KD: 100%; in dRYBP + PC KD: 100%. Efficiency of SCE reduction in SCE KD: 66%; in dRYBP + SCE KD: 62%. Efficiency of dKDM2 reduction in dKDM2 KD: 93%; in dRYBP + dKDM2 KD: 98%. Efficiency of PC reduction in PC KD: 62%; in dRYBP + PC KD: 68%. (**D**) Levels of H2Aub in the indicated KDs. The intensity of the bands corresponding to H2Aub and H2A was measured and quantified using Fiji imaging software calculating the different H2Aub/H2A ratios. H2Aub levels reduction (%) in dRYBP KD: 65%, in SCE KD: 95%, in dKDM2 KD: 56%, in PC KD: 5%, in dRYBP + SCE KD: 90%, in dRYBP + dKDM2 KD: 49%, in dRYBP + PC KD: 46%. Note that the decrease in H2Aub levels in dRYBP KD shown in (A) and (D) seem to be slightly different probably due to experimental conditions. Also note the efficiency of the indicated dsRNA-inactivation shown in (C). (**E**) Levels of H3K36me2 in the indicated KDs. Note levels of H3K36me2 decrease in dRYBP + dKDM2 KD in comparison to dKDM2 KD and the efficiency of the indicated dsRNA-inactivation shown in (C). (**F**) Levels of H2Bub in the indicated KDs. Note that levels of H2Bub increase in dRYBP + dBRE1 KD in comparison to dBRE1 KD and the efficiency of the indicated dsRNA-inactivation. dRYBP reduction in dRYBP KD: 93%; in dRYBP + dBRE1 KD: 98%. dBRE1 reduction in dBRE1 KD: 97%; in dRYBP + dBRE1 KD: 100%. Arrowheads indicate the bands corresponding to SCE. Arrows indicate the bands corresponding to dKDM2. Tubulin, H3, H2A and H2B were used as loading controls. Mock is GFP-dsRNA in all cases. To prove reproducibility of the results between 4–5 replicates of all the experiments were performed.

To test this, we analyzed histone modifications levels in S2 cells depleted of dRYBP, SCE, dKDM2, dBRE1 and PC (as a control) by RNAi-mediated knockdown (KD) (Figure 4). In dRYBP-KD cells, the levels of H3K36me2, H3K27me3, H2Aub, H2Bub and H3K4me were examined by Western Blot analysis using specific antibodies (Figure 4). dRYBP depletion caused a strong decrease in H2Aub and H3K4me and slight decrease in H3K36me2, whereas H3K27me3 and H2Bub remain unchanged (Figure 4A). Thus, dRYBP stimulates directly or indirectly H2Aub, H3K4me and H3K36me2.

Next, we analyzed H3K27me3 levels in S2 cells were either dRYBP, SCE, dKDM2 and PC (as a control) were inactivated. We found that in any of the analyzed cases H3K27me3 levels were affected (Figure 4B). This results suggest that dRYBP, SCE and dKDM2, all subunits of the RYBP-PRC1 complex variant, are not required for H3K27me3 levels and therefore for PRC2 activity and/or PRC2 recruitment.

Further, we analyzed H2Aub levels in S2 cells where either dRYBP and SCE or dRYBP and dKDM2 were individually or concomitantly inactivated (Figure 4D). Reduced levels of H2Aub were observed in the single knockdowns dRYBP-KD (55%; this reduction is not due to decreased SCE protein levels, Figure 4C), in SCE-KD (5%), and in dKDM2-KD (44%). This compares to reduction of H2Aub levels in the double knockdowns dRYBP + SCE-KD (10%) and dRYBP + dKDM2-KD (51%) (Figure 4D). We also checked H3K36me2 levels. Depletion of dRYBP (Figure 4A) has a weak effect on H3K36me2 levels while depletion of dKDM2 results, as previously described [11], in increased H3K36me2 levels (Figure 4E). Interestingly, the double knockdown dRYBP + dKDM2-KD (Figure 4E) strongly decreases H3K36me2 levels when compared to dKDM2-KD (this reduction is not due to increased dKDM2 protein levels, Figure 4C). Thus, dRYBP counteracts the repressor effect of dKDM2 through the modulation of the H3K36me2 levels.

Finally, we investigated whether dRYBP modulates dBRE1-dependent H2B monoubiquitylation. Depletion of dRYBP (Figure 4A and Figure 4F) does not have an effect on H2Bub while, as previously described [37] depletion of dBRE1 abolishes H2Bub (Figure 4F). Curiously, the simultaneous depletion of both dRYBP and dBRE1 results in increased levels of H2Bub compared to H2Bub levels in dBRE1-KD (Figure 4F, this reduction is not due to increased dBRE1 protein levels). Thus, dRYBP may counteract the activating function of dBRE1 by decreasing, directly or indirectly, H2Bub levels.

Taken together, the results from our investigation on the dRYBP modulation of post-translational modified histone levels (Figure 4), indicates that dRYBP stimulates H2A monoubiquitylation. We did not observe any effect of dRYBP on SCE-dependent H2A monoubiquitylation. We believe this is simply due to the fact that H2Aub is nearly completely absent in SCE depleted S2 cells. Furthermore, we have shown for the first time that inactivation of dRYBP, SCE and dKDM2 in S2 cells does not affect H3K27me3, suggesting that these proteins are not required for PRC2 activity. Moreover, it has been recently shown that non-canonical PRC1 complexes can be recruited to chromatin independently from H3K27me3 and suggested that H2Aub landmark established by PRC1 is responsible for PRC2 recruitment [13]–[16]. However, our results suggest that in *Drosophila* the repressive activity of PRC2 is independent from the PRC1 proteins dRYBP, SCE, dKDM2 and PC, reinforcing the classical hierarchy PcG complexes recruitment model [12],[14]. Our results also show that dRYBP slightly stimulates H3K36me2 levels but strongly suppresses dKDM2-mediated H3K36me2 demethylation suggesting that dRYBP alleviates dKDM2-mediated repression. Finally, these results show that dRYBP does not influence H2Bub levels but suppresses the dBRE1-mediated H2B monoubiquitylation, thus suggesting that dRYBP may alleviate dBRE1-mediated activation.

### A model for dRYBP function

**Figure 5:**
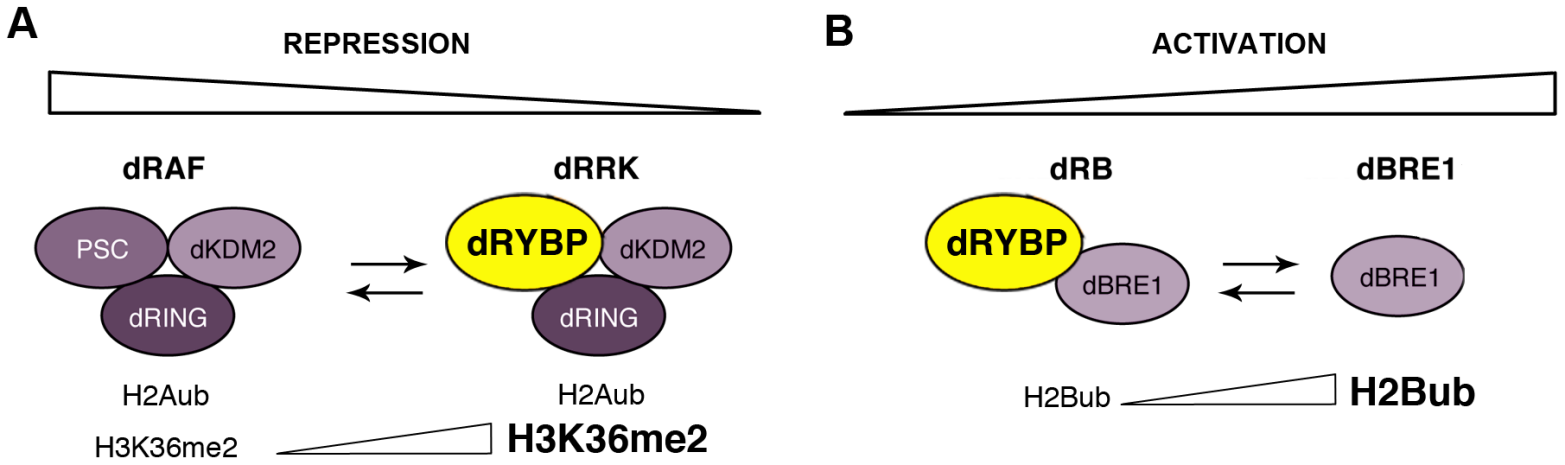
Hypothetical model for dRYBP function. (**A**) The dRRK complex (dRYBP + dRING + dKDM2) counteracts dRAF-mediated repression by increasing H3K36me2 levels and perhaps, decreasing H2Aub due to the absence of PSC. (**B**) The dRB complex (dRYBP + dBRE1) counteracts dBRE1-mediated activation by lowering H2Bub levels.

The biochemical interaction studies (Figure 2) propose the existence of the dRRK (composed of dRYBP, dRING/SCE, and dKDM2) and of the dRB (composed of dBRE1 and dRYBP) complexes. The composition of these putative complexes may include other subunits that are still to be determined. The mass spectrometric analysis did not detect interactions with any other of the PRC1 or PRC2 complexes proteins subunits such as PC, PSC, SU(Z)2, PH or even with PHO that was previously found, in over-expression experiments, to interact with dRYBP [20]. Neither detected interaction with RAD6, the only protein so far described to interact with BRE1 [7]. Our genetic interaction results (Figure 3) show that dRRK and dRB function to regulate homeotic gene expression and therefore these complexes are required for morphogenesis. Importantly, we show that dBRE1 is also involved in the process of morphogenesis mediated by the homeotic genes (Figure 3K). The results indicate that *dRYBP* enhances the repressor effect of *Sce* and also that *dRYBP* counteracts the *dkdm2* repressor effect. Our findings also show that *dRYBP* suppresses the activator effect of *dBRE1*. How does dRYBP control these antagonistic effects? We propose that dRYBP modulates modified histone levels to generate both a transcriptional repression state that is relatively weaker than the one promoted by dRAF complex and a transcriptional activator state that is relatively weaker than the one promoted by dBRE1. Thus, dRYBP epigenetically regulates gene expression through its ability to generate crosstalk between repression and activation: it promotes the alleviation of repression and the alleviation of activation of transcription. In this model (Figure 5), the interaction of dRYBP with SCE/dRING and dKDM2 generates the dRRK complex that, in turn, may exclude PSC from dRAF thereby impeding dKDM2 demethylase activity. The concurrent hypothetical decrease in H2Aub levels (due to the absence of PSC-E3 ubiquitin ligase) and increase in H3K36me2 levels causes a decrease in dRAF mediated transcriptional repression and generation of a comparatively lower state of transcriptional repression. Also in this model, the interaction of dRYBP with dBRE1 to form the dRB complex, may inhibit dBRE1 activity. The relatively lower levels of H2Bub will result in a relatively lower state of transcriptional activation. This model is supported by recent findings indicating that RYBP target genes expression present moderate levels of repression [9]. Perhaps the presence of dRYBP at specific *cis*-regulatory regions of target genes may serve to maintain different levels of expression in different cells or different parasegments (ps). For example, this system may help to control the expression of the homeotic *Ultrabithorax* protein in the ps5 or ps6 of the embryo or expression of the homeotic *Abdominal-B* gene in ps10, ps11 or ps12 [38]. Moreover, the ability of dRYBP to modulate both repression and activation may serve to provide epigenetic transcriptional plasticity that underlies the control of developmental transitions, homeostasis and pathological states.

## Materials and Methods

### *Drosophila* strains and handling

Flies *y^1^*, *Df(1)^w67c23^* were used as control. The PcG and trxG mutant alleles used were: *Sce^1^* and *Pc^3^* [35], *trx^E2^* [39], *dkdm2^KG04325^* [11], *dBre1^01640^* [40], *dBre1^kim1^* [41] and *dRYBP^1^* [20]. All crosses were performed at 25°C.

### Cell cultures and RNAi-mediated knockdowns

*Drosophila* S2 cells were cultured in Schneider's media (Invitrogen) supplied with 10% FBS and treated and incubated for 4 days with double-stranded RNA (dsRNA) as described [42]. dsRNA was synthesized using the Ambion Megascript T7 kit according to manufacturer's protocol. Primer sequences used to generate dsRNA directed against dRYBP were: 5′TTAATACGACTCACTATAGGGAGAGTGATCGAGGAGAACTTCTGG 3′, 5′ TTAATACGACTCACTATAGGGAGAGCTGTCGTTGCTCTCGCTGAA 3′. Primer sequences used for dsRNA directed against SCE, dKDM2 and PC were previously described [11], dBRE1 [41]. After 4 days, histones were isolated from samples by acid extraction [11] and used for protein detection. When required cells were treated with proteasome inhibitors as described in [43].

### Plasmids, GST-pulldowns and Co-immunoprecipitations

dRYBP and dRYBPΔNZF [20] cDNAs were cloned into pGEX2TKN, a derivate of pGEX-2TK (Pharmacia) using the primers: 5′ TTTCATATGATGGACAAGAAATCCTCGCCG 3′, 5′ TTTCATATGGCCTCCGGATCACGGCATGGC 3′ and 5′TTTTCTAGACTAACTCCGGCTGTCGTTGCT 3′. Proteins were expressed as glutathione S-transferase (GST) fusion proteins and purification and GST-pulldowns were performed as described [44]. Co-IPs were performed as previously described [11].

### Western Blot analysis

Protein samples were separated on 8, 12, 15 or 18% SDS-PAGE gel and WB were performed following standard procedures. The primary antibodies used were: Mouse: α-Ub (1∶1000) (Millipore clone FK2), α-Tubulin (1∶8000) (Sigma), α-H2Aub (1∶250) (Merck, clone E6C5), α-H2Bub (Millipore, clone 56), (1∶1000); Rabbit: α-dRYBP (1∶250) [19], α-PC (1∶500) [11], α-PH (1∶500) [11], α-dBRE1 (1∶1000) [41], α-H2A (1∶500) (Abcam 13293), α-H2B (1∶2000) (Upstate 07-371), α-H3 (1∶2000) (Abcam 1791), α-H3K36me2 (1∶2000) (Upstate 07369), α-H3K27me3 (1∶1000) (Upstate 07-449), α-H3K4me3 (1∶1000) (Active Motif 39159) α-H3K4me (1∶500) (Abcam ab8895); Guinea pig: α-PSC (1∶250) [11], α-SCE (1∶500) [11], α-dKDM2 (1∶250) [11], α-E(Z) (1∶500) [11]. AP (Alkaline Phosphatase) coupled secondary antibodies were used (Sigma-Aldrich).

### Mass spectrometric analysis

*Drosophila* nuclear protein extracts from 0-12h wild type embryos were prepared as previously described [45] and incubated with two different affinity purified antibodies (dRYBP #194 and dRYBP #195) directed against dRYBP [19] previously coupled to sepharose A beads (GE Healthcare). After incubation, beads were extensively washed with buffers containing either 400 mM or 800 mM KCl and 0.1% NP-40. dRYBP immunopurified fractions were resolved by SDS-PAGE and visualized by silver staining following standard protocols [46]. Proteins present in bands excised from the gel were identified by nanoflow LC-MS/MS at the Proteomics Center, Erasmus Medical Center, Rotterdam.

### Cuticle preparation

Flies were dissected and mounted as previously described [22]. Images were generated using a Zeiss CCD microscope and processed using Adobe PhotoShop CS5.

## Supporting Information

Figure S1 **Analysis of dRYBP binding to ubiquitin and ubiquitylated proteins from S2 cell extracts untreated with proteasomal inhibitors (Lactacystin and MG132).** (**A**) Western Blot using α-dRYBP and α-Ub antibodies of S2 cells protein extracts untreated (Mock) and treated with proteasome inhibitors (+MG-132+ Lactacystin). Note that α-dRYBP detects 17 kDa and 25 kDa bands and that α-Ub detects a 25 kDa band. (**B**) Pulldown assay performed using S2 cell extracts (Input) with GST-protein (Mock) and fusion proteins dRYBP-GST and dRYBPΔNZF-GST. Proteins were analyzed by immunoblotting with α-Ub antibody. Note levels of ubiquitylated proteins are very low, including the Input.
(PDF)

Figure S2 **dRYBP does not interact biochemically with PSC, PC, PH and EZ.** (**A**) Drosophila embryonic wild type nuclear extracts (Input) or pre-immune serum (Mock) were immunoprecipitated using α-PSC, α-PC, α-PH and α-E(Z) antibodies. Eluted proteins were resolved by SDS-PAGE and analyzed by Western Blot for dRYBP, SCE, dKDM2, PSC, PC, PH and E(Z) detection. Note dRYBP protein does not interact with any other protein. Arrowheads point to the corresponding protein bands detected with the indicated antibodies (other bands may be non-specific or correspond to modified proteins).
(PDF)
