# Supplementary figures and images for: dRYBP Counteracts Chromatin-Dependent Activation and Repression of Transcription
Source: PLoS One. 2014 Nov 21;9(11):e113255. doi: 10.1371/journal.pone.0113255 (PMC4240632; doi:10.1371/journal.pone.0113255)

Figure S1. Fereres, S. *et al.*

**A**

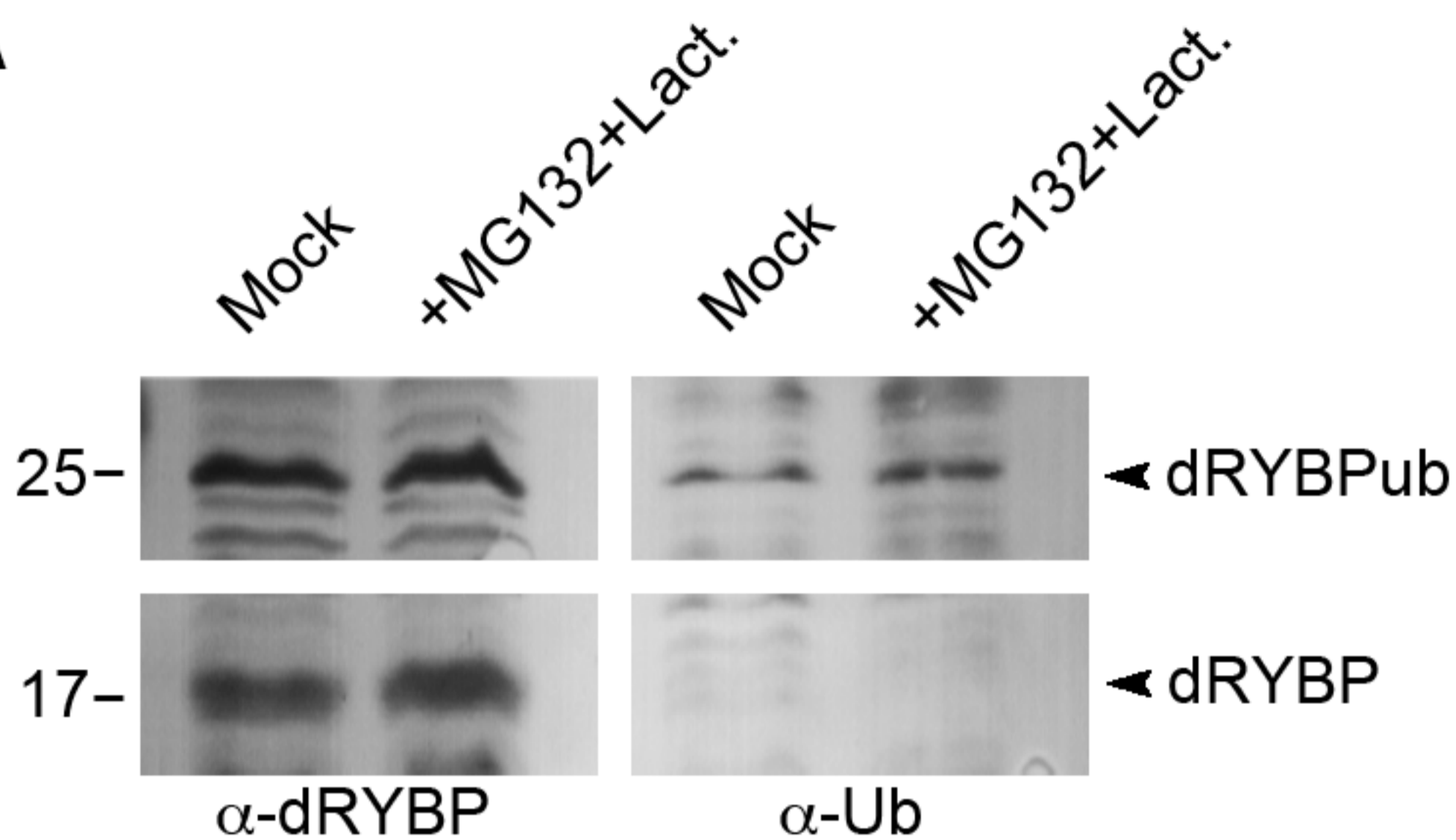

**B**

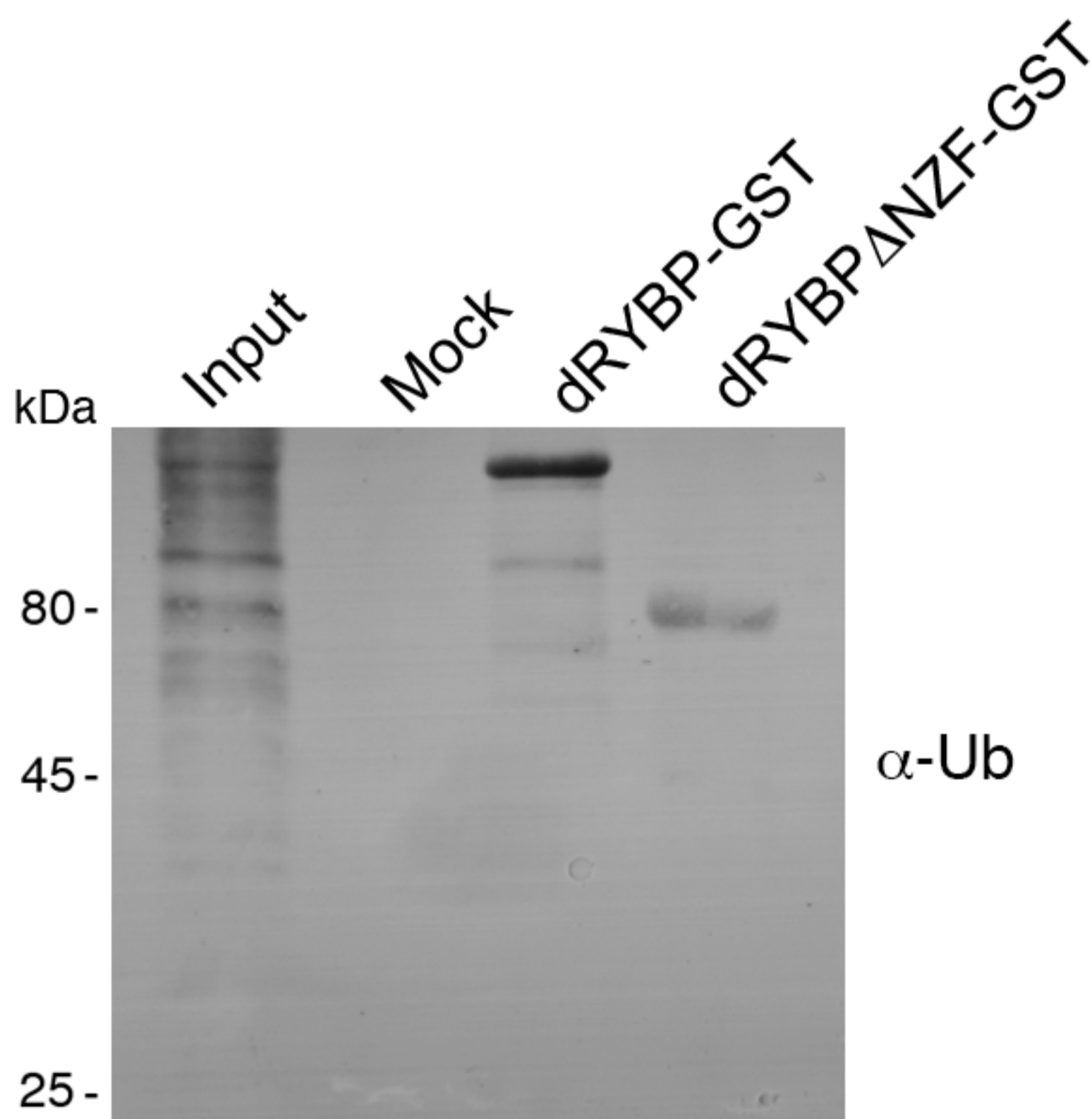

Supplement: Figure S1 — Analysis of dRYBP binding to ubiquitin and ubiquitylated proteins from S2 cell extracts untreated with proteasomal inhibitors (Lactacystin and MG132). (A) Western Blot using α-dRYBP and α-Ub antibodies of S2 cells protein extracts untreated (Mock) and treated with proteasome inhibitors (+MG-132+ Lactacystin). Note that α-dRYBP detects 17 kDa and 25 kDa bands and that α-Ub detects a 25 kDa band. (B) Pulldown assay performed using S2 cell extracts (Input) with GST-protein (Mock) and fusion proteins dRYBP-GST and dRYBPΔNZF-GST. Proteins were analyzed by immunoblotting with α-Ub antibody. Note levels of ubiquitylated proteins are very low, including the Input. (PDF) [file pone.0113255.s001.pdf]

Figure S2. Fereres, S. *et al.*

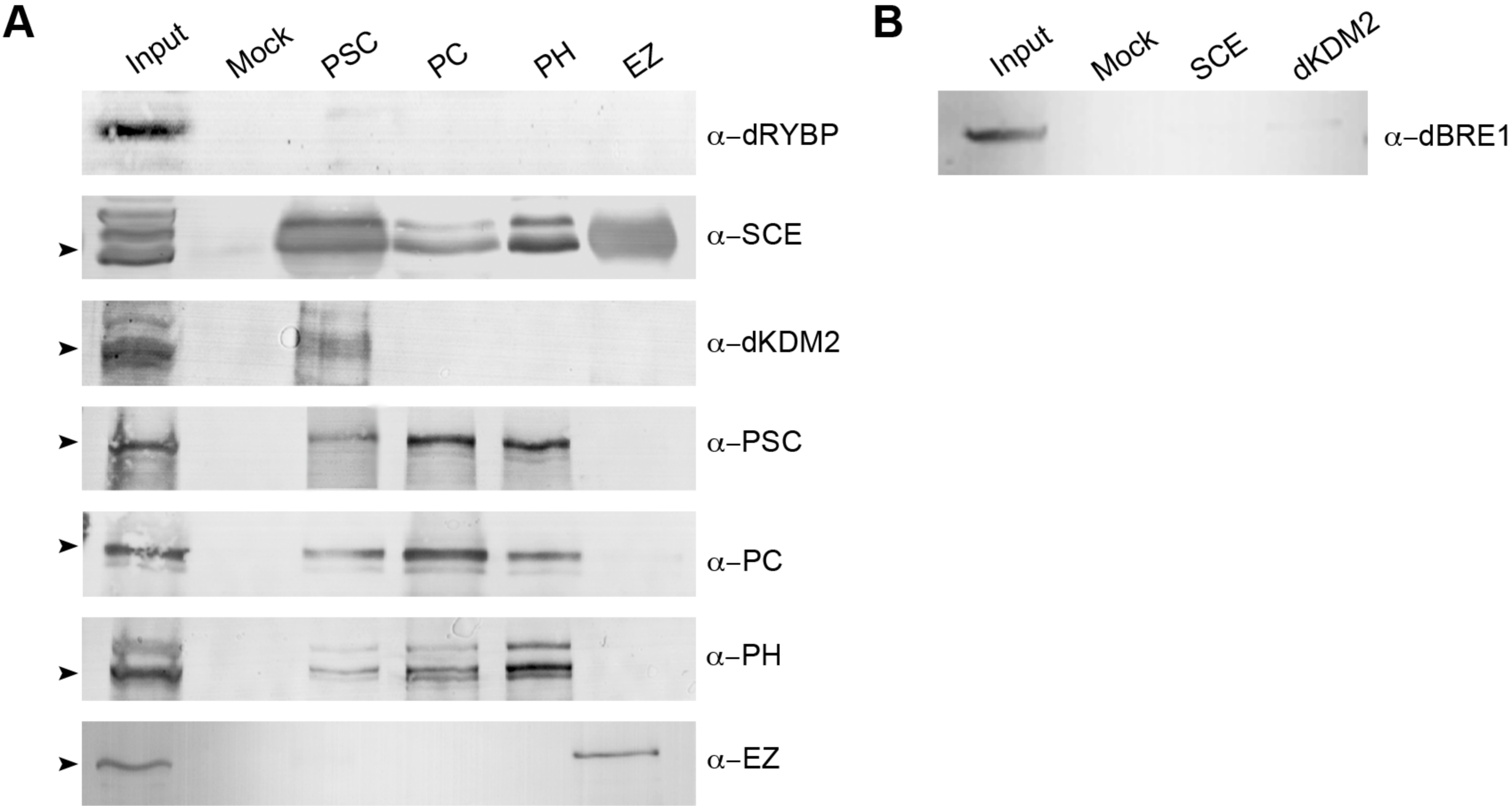

Supplement: Figure S2 — dRYBP does not interact biochemically with PSC, PC, PH and EZ. (A) Drosophila embryonic wild type nuclear extracts (Input) or pre-immune serum (Mock) were immunoprecipitated using α-PSC, α-PC, α-PH and α-E(Z) antibodies. Eluted proteins were resolved by SDS-PAGE and analyzed by Western Blot for dRYBP, SCE, dKDM2, PSC, PC, PH and E(Z) detection. Note dRYBP protein does not interact with any other protein. Arrowheads point to the corresponding protein bands detected with the indicated antibodies (other bands may be non-specific or correspond to modified proteins). (PDF) [file pone.0113255.s002.pdf]
